# Numerical Study on the Effect of Matrix Self-Heating on the Thermo-Visco-Plastic Response of Continuous Fiber-Reinforced Polymers under Transverse Tensile Loading

**DOI:** 10.3390/polym14101941

**Published:** 2022-05-10

**Authors:** Ruben D. B. Sevenois, Pei Hao, Wim Van Paepegem, Francisco A. Gilabert

**Affiliations:** 1 Department of Materials, Textiles and Chemical Engineering, Faculty of Engineering and Architecture, Ghent University, Technologiepark 46, B-9052 Ghent, Belgium; pei.hao@ugent.be (P.H.); wim.vanpaepegem@ugent.be (W.V.P.); fran.gilabert@ugent.be (F.A.G.); 2 SIM M3 Program, Technologiepark 48, 9052 Ghent, Belgium

**Keywords:** polymer-matrix composites (PMCs), thermomechanical properties, non-linear behaviour, multiscale modeling, representative volume element (RVE)

## Abstract

The recyclability and improved suitability for high-volume production make fiber-reinforced thermoplastic polymers (FRP) attractive alternatives for the current thermoset-based ones. However, while they are more ductile than their thermoset counterparts, their behavior is also more susceptible to environmental conditions such as humidity, temperature, and strain rate. The latter can trigger self-heating and thermal softening effects. The role of matrix self-heating in FRP subjected to transverse loading is investigated using micromechanical modeling. Particularly, the effect of self-heating, strain rate and conductivity of the fiber-matrix interface is illustrated. It is shown that local heating of the matrix is dominant for the homogenized behavior of the material. Although the global homogenized temperature increase is limited, local thermal softening can induce premature failure. It is shown that the effect of thermal softening can be more prominent with increasing volume fraction, increasing strain rate, and lower interface conductivity.

## 1. Introduction

Fiber-reinforced polymers (FRP) with thermoplastic matrix material offer, compared to thermoset FRP, better recyclability and improved suitability for high-volume production processes. These properties make them an attractive alternative for the current thermoset-based FRP in high strength and low weight applications for both low volume (air, space, wind energy) and high-volume applications (e.g., automotive, transport). However, they are more ductile than their thermoset-based equivalent, their behavior is also more susceptible to environmental conditions, such as humidity and temperature. Moreover, their inherent rate-dependency adds difficulties in the matrix characterization due to a lack of understanding of the relaxation process of polymer chains. Additionally, most thermoplastics are semi-crystalline polymers where complex morphological amorphous and crystalline phases are involved. Viscous dissipation, which occurs at an elevated strain rate, can naturally trigger self-heating and the associated thermal softening. Considering the competition between plastic deformation, associated heat generation, and thermal diffusion, coupled thermomechanical analysis of these materials is necessary to enable the use of thermoplastic materials in FRP.

The constitutive behavior of FRP can either be determined experimentally or estimated using advanced simulations. When a multitude of variables are involved (e.g., temperature, humidity, strain rate, pressure) an experimental campaign including all possible combinations of parameters quickly becomes too expensive to execute in terms of time, money, and resources. As an alternative, advanced simulation methods based on the micromechanics of the material can provide at least a qualitative prediction of the material response. This type of simulation environment enables investigating the effect of multiple parameters, including environmental conditions, on material behavior, at a limited added cost. In this way, the most promising combinations of fibers and matrices, for the given design conditions can be chosen for a thorough quantitative experimental assessment.

Micromechanical simulation of FRP usually consists of the analysis of a Representative Volume Element (RVE) in which the fibers, matrix, and the conductivity of the fiber-matrix interface are treated as individual entities. In the RVE, each constituent is assigned its individual constitutive behavior. Then, the combined response of the constituents and their interaction in the RVE, subject to several boundary conditions, is homogenized to the macroscale [1]. Crucial for these simulations is the definition of the constituent behavior, which should be accurate and representative of the material at the microscale. Otherwise, as shown by Chevalier et al. [2], erroneous conclusions can be drawn.

Computational micromechanics has been used to simulate the mechanical response of the FRP undergoing different conditions by varying the temperature and strain rates, as well as investigating the effect of the constituent materials [3]. Micromodels of UD FRP are primarily used to investigate the transverse and shear behavior of the material because the properties of the matrix and the conductivity of the fiber-matrix interface dominate the macroscale UD response. Melro et al. [4], Huang et al. [5], and Garoz et al. [6] focused on the generation of periodic microstructures with random fiber arrangements. They concluded that a model size containing 30 to 50 fibers is sufficient to represent the homogenized behavior. Sharma and Daggumati [7], Liu and Li [8], and Wan et al. [9] investigated the initial failure location and damage evolution under transverse loading. The works of Yan and Ran [10], Asp et al. [11], Hobbiebrunken et al. [12], and Moraleda et al. [13] investigate the effect of the strength of the interface on the damage evolution. Wang et al. [14] investigated the effect of friction after fiber-matrix debonding under transverse compression. Maligno et al. [15], Totry et al. [16,17], Segurado and Llorca [18], and Vaughan and McCartney [19,20] also included the effect of thermal residual stress generated during the production process. The influence of strain rate is investigated by Sao et al. [21] and Shafiei et al. [22]. Arteiro et al. [23] investigated the effect of neighboring plies on the damage development inside a central ply. Jordan et al. [24], Sato et al. [21], and Bai et al. [25] included the effect of temperature on the macroscale behavior. This is done by including a temperature dependency in the constitutive equations of the matrix material.

In these models, the fibers (typically glass or carbon) are modeled as linear elastic material, while the constitutive behavior of the matrix and/or the interface varies depending on the type of matrix, type of deformation, and environmental conditions. For example, Bai et al. [25] use a paraboloidal yield criterion in combination with a thermo-visco-plastic hardening law to include both the effect of strain rate and temperature. Sharma and Daggumati [7] use a linear Drucker–Prager plasticity model with a ductile damage criterion for the matrix. The choice of the specific constitutive behavior for the matrix is usually based on the experimental evidence from tensile or compressive tests on the bulk matrix. However, several authors have already presented evidence that the bulk matrix behavior might not be representative of the behavior of the matrix confined in small resin pockets between fibers [2,26]. It seems that the local yield stress of the matrix appears to be higher, resulting in a more brittle behavior compared to the bulk. In contrast, Verschatse et al. [27] found that the yield stress of microfiber epoxy matrix is lower than the yield stress of the bulk material. The difference might be caused by the different loading conditions applied. Verschatse et al. [27] applied tensile loading, while Chevalier et al. [2] applied compressive load. Other differences might be caused by a different arrangement of the polymer chains, guided by the fiber direction, or different crystal structures (in the case of thermoplastic (TP) matrices) initiated by the fiber type [28]. In general, the modeling of a matrix material compatible with micromechanical simulations needs to be carefully considered.

As aforementioned, the influence of several matrix types with thermal, viscous, and plastic constitutive response has been investigated previously. However, it is known that polymeric materials generate heat when visco-elastic and visco-plastic deformations occur. This phenomenon, also known as self-heating, is primarily important at high strain rates under monotonic loading or at high frequencies under cyclic loading [29,30]. Since the local strain rate in matrix pockets might be significantly different than the macroscopically applied strain rate, the question of whether localized self-heating plays an important role in the macroscopic response of the FRP remains open.

In this work, the role of matrix self-heating in FRP subjected to transverse loading at the micro- and macroscale is investigated. Particular attention is paid to the effect of self-heating, strain rate, and the conductivity of the fiber-matrix interface. For this, an RVE with a microstructure referring to a UD Carbon/PA6 FRP is used. The fibers are modeled as transverse isotropic linear-elastic materials. The matrix is modeled based on the polymer yield kinetics phenomenology recently developed in a thermo-visco-plastic constitutive law for semi-crystalline polymers, which includes self-heating [31]. The interface between fibers and matrix is included as a conductive interface with cohesive behavior. The role of self-heating is investigated by applying several macroscopic strain rates to the RVE. The amount of local self-heating and its dispersion through conduction is determined. At the same time, the generation of self-heating as a function of the fiber volume fraction and the conductivity of the fiber-matrix interface is studied and quantified.

This paper is structured as follows. In Section 2, the RVE model, its generation, and the application of the periodic boundary conditions are explained. In Section 3, the material constitutive relationships, including the thermal properties of the fibers, matrix, and fiber-matrix interface are given as well as the determination of the material parameters. Section 4 presents a study on the effect of self-heating and the conductivity of the fiber-matrix interface in relation to the applied strain rate and fiber volume fraction. Finally, Section 5 highlights the main conclusions.

## 2. RVE Modeling

An RVE model is required in order to study the effect of matrix self-heating on the micromechanical behavior of a UD composite ply. The elements necessary to construct this RVE are the geometry, the boundary conditions, and the appropriate material constitutive relationships. The latter is discussed in the following section.

**Figure 1:**
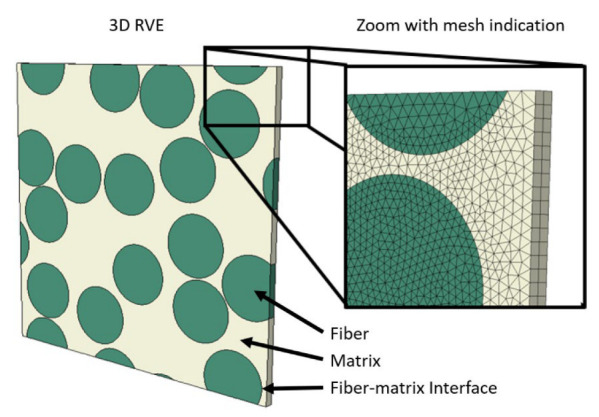
Periodic RVE with 16 fibers and a fiber volume fraction of 50%.

The selected composite material for this research corresponds to a UD Carbon/PA6 FRP represented by randomly positioned cylinders embedded in a polymer matrix assuming periodic boundary conditions. This geometry is illustrated in Figure 1 for a model with 16 fibers and a fiber volume fraction of 50%.

The position of the fibers is generated using a random generator previously used for molecular dynamics simulation of two-dimensional cohesive granular materials [32]. The fiber diameter is set to 7 µm. This is a regular size for carbon fibers. Fibers and matrix are independently meshed using C3D6T wedge elements [33]. A mesh convergence study is carried out to ensure that the elements are sufficiently small. This resulted in an approximate element size of 0.4 µm. The fiber matrix-interface is modeled using a surface-to-surface contact definition accounting for thermal conductivity and cohesive behavior. The particular values used for the thermal conductivity and cohesive behavior are given in Section 3.

Mechanical Periodic Boundary Conditions (MPBC) are assigned according to the relative formulation with Node-to-Node coupling by Garoz et al. [34]. Through these MPBCs, a mechanical load in the transverse direction is applied while Poisson’s contraction is allowed. In a similar fashion as for the MPBCs, Thermal Periodic Boundary Conditions (TPBC) are introduced as follows:

(1) $$\theta^{x,min}-\theta^{x,max}=\Delta \theta_{x}\;\theta^{y,min}-\theta^{y,max}=\Delta \theta_{y}\;\theta^{z,min}-\theta^{z,max}=\Delta \theta_{z}$$

where θ is the temperature on the nodes on opposite surfaces in the x, y and z-direction. These equations are applicable, regardless of whether the RVE is used to predict the thermal conductivity or the thermomechanical response. For the prediction of thermal conductivity, a temperature difference is applied by setting $\Delta \theta_{x}$, $\Delta \theta_{y}$, $\Delta \theta_{z}$ to a non-zero value. This would induce a heat flux between the nodes on the opposite surfaces from which thermal conductivity can be obtained. For thermomechanical response, a specific heat flux between parallel sides of the RVE is not desired. Therefore $\Delta \theta_{x}=\;\Delta \theta_{y}=\Delta \theta_{z}=0$, ensuring that opposite nodes have the same temperature. The latter is key to generating a meaningful constitutive behavior. The initial temperature of the RVE is set to 23 °C.

## 3. Material Constitutive Behavior

The thermomechanical constitutive behavior of the fibers, matrix, and fiber-matrix interface are discussed in this section, together with the initial values for the material parameters. Some of these initial parameters are varied in subsequent sections to study the effect on the homogenized response. Any adaptation of specific parameters is mentioned in the respective section.

### 3.1. Fiber Material

It is assumed that the carbon fibers follow a linear elastic and transversely isotropic response. The properties for the mechanical and thermal constitutive behavior are adopted from Arteiro et al. [26] and Dong et al. [35,36], respectively. The properties are given in Table 1.

### 3.2. Matrix Material

The matrix material is modeled using the Unified Semi-Crystalline Polymer (USCP) constitutive model based on [31] and further developed in [37]. This model has an improved representation of the double-yield phenomenon considering a temperature-dependent visco-plastic response, including self-heating and thermal softening effects.

**Figure 2:**
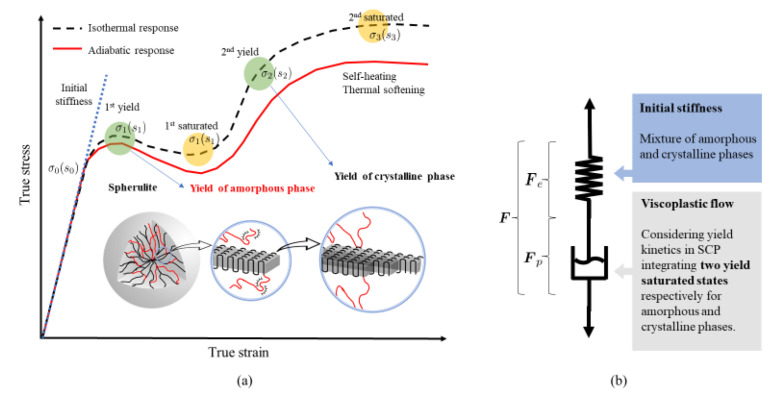
Schematic diagram of the morphological transformation of molecular structure in SCP with (**a**) corresponding stress-strain curve and (**b**) rheological representation.

Figure 2 schematically presents the stress-strain response of a typical SCP composed of elementary spherulites. The associated rheological representation is depicted with a newly developed visco-plastic dashpot that integrates two athermal strengths, $s_{2}$ and $s_{3}$, corresponding to the saturated states of the amorphous and crystalline phases, respectively. Owing to the complex yield kinetics of SCPs [31], two yield points correlated to the double kink release process in the amorphous phase and the crystal slips in the crystalline phase are derived.

The amorphous phase is characterized following the parameter identification procedure of a modified Boyce–Park–Argon model described in [38]. Additionally, three material parameters ${\bar{\varepsilon }}_{c}$, $s_{3},$ and $h_{3}\;$are introduced to characterize the stress contribution of the crystalline phase. The coupled thermo-mechanical response is implemented in a finite strain kinematic framework. A brief overview of the constitutive equations for the matrix behavior is given in the following.

The Cauchy stress tensor is calculated using the linear spring in terms of the elastic deformation gradient $F_{e}$, given by:

(2) $$\sigma =\frac{1}{det\left.[F_{e}\right]}\left(\lambda tr\left[h\right]I+2\mu h\right),$$

where λ and μ are the Lamé constants, I is the second order identity tensor, and h is the Hencky strain tensor.

To update the elastic deformation gradient, the plastic deformation gradient $F_{p}$ is required. $F_{p}$ is obtained using the inelastic rate of deformation $D_{p}$ by integrating $\dot{F_{p}}=\left(F_{e}\right){\;}^{-1}D_{p}F$. The prescribed form is expressed as:

(3) $$D_{p}=\dot{\bar{\varepsilon }}N$$

where N is the direction tensor and $\dot{\bar{\varepsilon }}$ is the constitutive effective plastic strain rate. The plastic flow follows a thermally activated visco-plastic law given by [31]:

(4) ε¯˙=ε0˙exp−Asθ1−32σ′:σ′sm

where $\dot{\varepsilon_{0}}$, m and A are the rate-dependent sensitivity parameters, which determine the first peak yield stress at different strain rates. θ is the absolute temperature. $\sigma^{\prime }$ is the deviatoric part of the stress tensor. s is the athermal strength initiated with a value [31]:

(5) $$s_{0}=\sqrt{3}\frac{8.5^{-1/m}}{\left(1-v\right)}\frac{E\left(\theta \right)}{2\left(1+v\right)}$$

Two representative athermal strengths in the saturated states of the amorphous and the crystalline phases are considered in the evolution of s as follows:

(6) $$\dot{s}=H_{1}\left(\bar{\varepsilon }\right)\;\left(1-\frac{s}{s_{1}}\right)\dot{\bar{\varepsilon }}+H_{2}\left(\bar{\varepsilon }\right)\;\left(1-\frac{s}{s_{2}}\right)\dot{\bar{\varepsilon }}+H_{3}\left(\bar{\varepsilon }\right)\;\left(1-\frac{s}{s_{3}}\right)\dot{\bar{\varepsilon }}$$

A series of smooth Heaviside-like functions is formulated to connect the yielding stages occurring in each phase [37]:

(7) $$H_{1}\left(\bar{\varepsilon }\right)=-h_{1}\left\{\tanh\left(\frac{\bar{\varepsilon }-{\bar{\varepsilon }}_{p}}{f{\bar{\varepsilon }}_{p}}\right)-1\right\}$$

(8) $$H_{2}\left(\bar{\varepsilon }\right)=h_{2}\left\{1-\tanh\left(\frac{\bar{\varepsilon }-{\bar{\varepsilon }}_{p}}{f{\bar{\varepsilon }}_{p}}\right)\tanh\left(\frac{\bar{\varepsilon }-{\bar{\varepsilon }}_{c}}{f{\bar{\varepsilon }}_{c}}\right)\right\}$$

(9) $$H_{3}\left(\bar{\varepsilon }\right)=h_{3}\left\{1+\tanh\left(\frac{\bar{\varepsilon }-{\bar{\varepsilon }}_{c}}{f{\bar{\varepsilon }}_{c}}\right)\right\}$$

To capture the experimental observation of self-heating, a thermomechanical coupling analysis is developed. The heat balance equation considering the associated plastic dissipation is included. Assuming constant thermal specific heat $c_{p}$ and thermal conductivity *k*, the heat balance equation is written as:

(10) $$\rho \;c_{p}\frac{\partial \theta }{\partial t}=\sigma \;:\;F_{e}D_{p}{\left(F_{e}\right)}^{-1}+\nabla \cdot \left(k\frac{\partial \theta }{\partial x}x\right)$$

Meanwhile, the thermal softening effect is naturally captured by taking into account the temperature dependency of the current elastic modulus. A logarithmic regression between the current and reference temperatures $\theta_{\mathrm{ref}}$ is adopted via a material constant β, according to Poulain et al. [38].

(11) $$E\left(\theta \right)=\frac{E_{\mathrm{ref}}}{10^{\beta \left(\theta -\theta_{\mathrm{ref}}\right)}}$$

**Figure 3:**
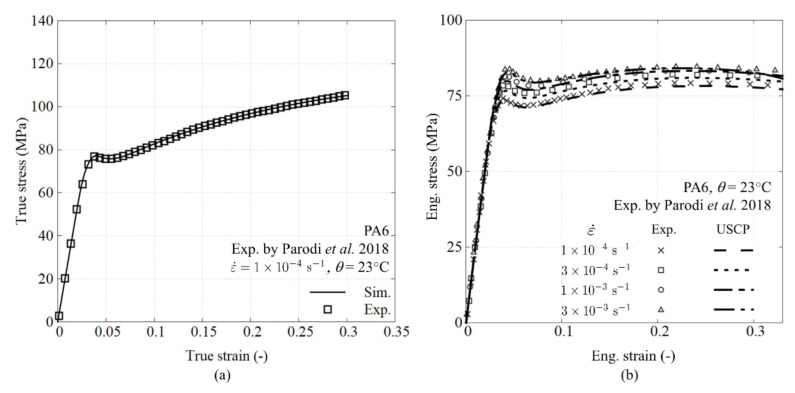
USCP model considering two saturated states (**a**) fitting of crystalline related parameter and (**b**) model validation using dog-bone test.

Figure 3 plots the results from model calibration and the corresponding validation process. To calibrate the intrinsic material behavior, attention should be paid to the conversion of the true stress-strain curve from the engineering one. The material parameters associated with PA6 are determined from the experimental results of Parodi et al. [39]. The amorphous related parameters are identified using an in-house developed optimization script based on a modified Nelder–Mead algorithm with a baseline stress-strain curve at the lowest strain rate (i.e., $\dot{\varepsilon }=1\times 10^{-4}s^{-1}$) [37,40]. First yield stress at a higher strain rate is supplied to determine the rate-sensitive parameters. The constitutive behavior of the matrix is included as a user subroutine in the finite element solver. The result after the fitting process for crystalline-related parameters is shown in Figure 3a. The calibration is based on a single element simulation, whereas the validation is performed on a real-scale dog-bone simulation. Figure 3b shows the validation of the experimental results and predictions of PA6 with a good agreement. Here, the engineering stress and strain are processed in the same way as the experimental tests. The engineering stress is calculated using the total pulling force and the initial cross-section. The strain is obtained with a virtual extensometer. The properties are summarized in Table 2.

### 3.3. Fiber-Matrix Interface

The fiber-matrix interface is considered as a zero-thickness conductive surface between the fiber and the matrix. This is implemented using the built-in general cohesive contact available in the FEM software. Compared to conventional approaches where cohesive elements are explicitly placed between matrix and fibers, the adopted surface-based formulation avoids unnecessary degrees of freedom (DOFs) without requiring a conformal mesh between fibers and matrix. Moreover, the formulation can be easily enriched with damage to model fiber-matrix debonding, which is planned for use in future work. Although it is known that the conductivity of the fiber-matrix interface can be different than either matrix or fiber conductivity [41,42,43,44], this value is initially set equal to the thermal conductivity of the pure matrix.

## 4. Results and Discussion

In this section, the effects of volume fraction, self-heating, strain rate, and interface conductivity on the thermomechanical behavior of the material are numerically investigated by varying the parameters controlling the aforementioned properties. Before this can be reliably done, however, the optimum size of the RVE must be verified.

### 4.1. Optimal RVE Size for Reliable Coupled Thermomechanical Analysis

**Figure 4:**
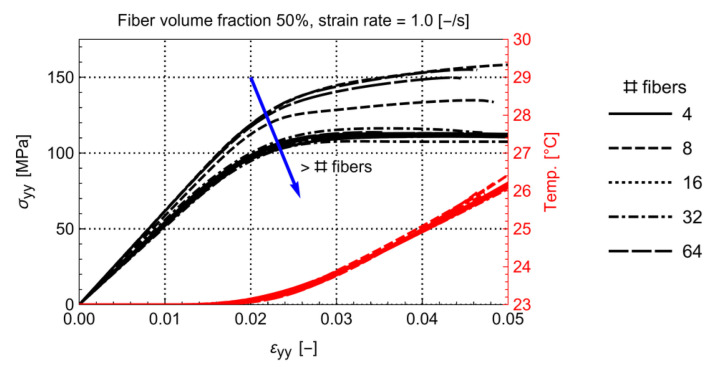
Homogenized stress-strain response for the models with 50% volume fraction and different amounts and arrangements of fibers.

Since previous works did not include TPBCs and a self-heating-enabled matrix model, the optimum size of the RVE (30 to 50 fibers [4,5,6]) must be re-evaluated. This is done by keeping the basic material properties for the RVE constant (described in Section 3) while multiple RVEs with different volume fractions and number of fibers are generated. Five different random arrangements were generated with four volume fractions (30%, 40%, 50%, 60%) and five amounts of fibers (4, 8, 16, 32, 64). This results in a total of 100 models which were mechanically loaded with a strain rate of $1.0\;s^{-1}$ This strain rate is chosen such that a moderate amount of heating would be generated inside the matrix. This is key to ensuring a fully thermal coupled regime [31]. The models are strained up to 5%. This is a high transverse strain for UD composites. Upon comparing experimental results, the failure strain is limited to 2.5% for a dry-conditioned C/PA6 at room temperature and quasi-static strain rate [28]. Based on this, one could argue that any result beyond 2.5% strain is irrelevant. However, under the influence of temperature and humidity, the matrix can become softer, which could result in an increased strain at failure. To anticipate this, 5% of transverse strain is considered a reasonable value. To determine the minimum number of fibers, the homogenized stress-strain and temperature-strain curves for each volume fraction are compared amongst each other. This is illustrated in Figure 4 for the volume fraction of 50%. It can be seen that, by increasing the number of fibers, the homogenized stress-strain response of the RVEs tends towards the lower stress bound. From 32 fibers and higher, the scatter in the stress-strain curves is sufficiently small.

As such, it is concluded that a minimum amount of 32 fibers is sufficient to result in a converged homogenized behavior.

Another observation is that the double yield phenomenon present in the matrix material, Figure 3, is obscured in the homogenized behavior of the RVE. The latter could be an issue for works where the matrix properties in an RVE are reverse engineered from macromechanical experimental testing of the composite material, as this behavior, if it should occur, would not be visible in these curves.

In the next sections, the influence of volume fraction, strain rate, self-heating, and interface conductivity is studied in detail. For this, it is not necessary to run all variations on each of the remaining 40 models (32 fibers and up) since these would give similar results. Therefore, only one representative RVE with 32 fibers is selected for each volume fraction.

### 4.2. Effect of Volume Fraction

**Figure 5:**
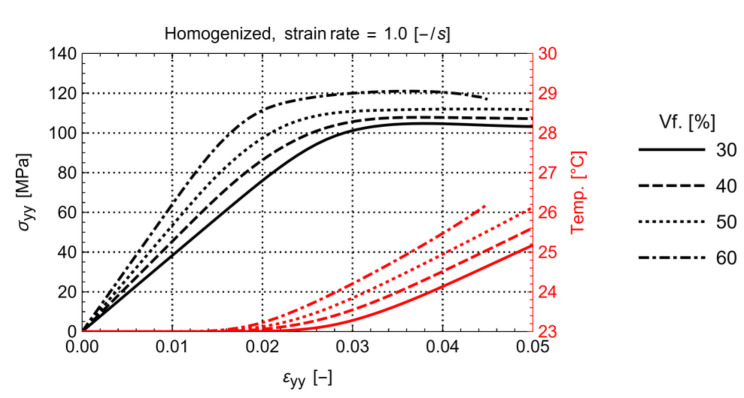
Homogenized stress-strain and temperature-strain with volume fraction.

The influence of volume fraction on the homogenized stress-temperature-strain for a load rate of $1.0\;s^{-1}$ is illustrated in Figure 5. It can be concluded that an increasing fiber volume fraction results in a higher stiffness of the material as well as higher yield stress and lower yield strain. The latter is generally expected.

**Figure 6:**
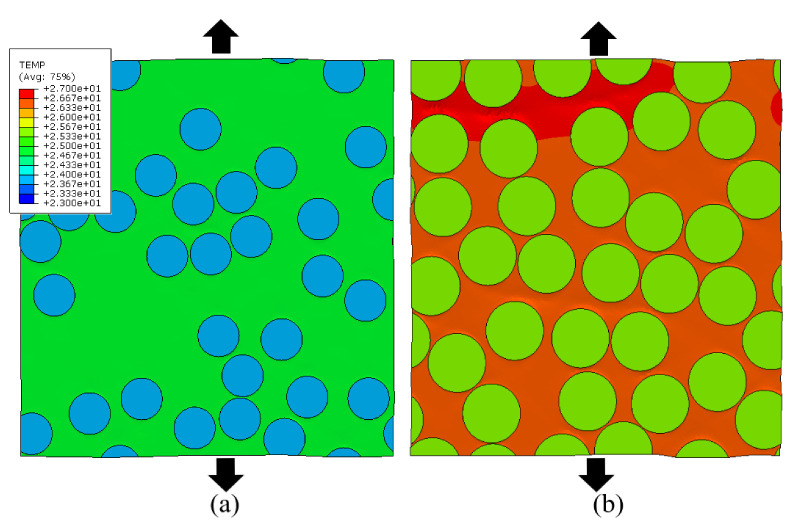
Temperature distribution for the RVEs with Vf. = 30% (**a**) and 60% (**b**) at a *ε* = 0.043 and $\dot{\varepsilon }$ = 1 s^−1^.

What might be surprising is that the temperature rise in the RVE, although limited for the strain rate of $1.0\;s^{-1}$, increases with higher fiber volume fraction. To result in an average increased temperature with increased fiber volume, the remaining matrix must be exponentially heating up. This indicates a significant increase in plastic dissipation in the matrix material for the same homogenized strain level. The latter can be seen in Figure 6 where the temperature distribution for the RVEs with 30% and 60% volume fraction at the same total strain is shown.

Note that both the matrix and the fiber temperature are higher for the volume fraction of 60%, indicating that the heat transfer from matrix to fibers is occurring as well. Clearly, the amount of self-heating, since it is related to the rate-dependent plastic dissipation influencing the matrix stiffness, has an effect on the global stress strain behavior at multiple strain rates.

### 4.3. Effect of Self-Heating and Strain Rate

**Figure 7:**
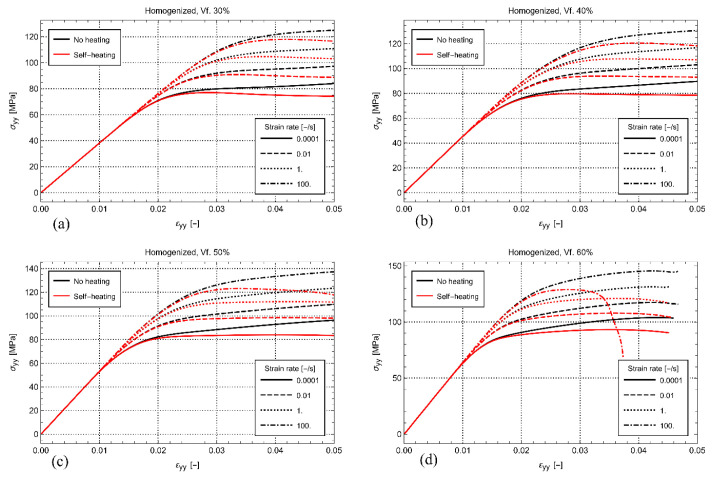
Homogenized stress-strain for the fiber volume fraction of 30% (**a**), 40% (**b**), 50% (**c**), and 60% (**d**) at several strain rates.

To study the influence of self-heating, a comparison is made between the cases where self-heating is disabled and enabled. With no self-heating, Equation (10) is deactivated, which effectively results in an RVE where the temperature remains constant at 23 °C during the entire loading. At the same time, to study the influence of strain rate, the global strain rate on the RVEs is varied between $10^{-4}\;s^{-1}$, $10^{-2}\;s^{-1}$, $1\;s^{-1}$, and $100\;s^{-1}$. Figure 7 shows the resulting homogenized stress-strain behavior for all the studied volume fractions. This figure clearly shows that the self-heating of the matrix softens the visco-plastic response. Generally, the amount of softening increases with increasing volume fraction and increasing strain rate.

**Figure 8:**
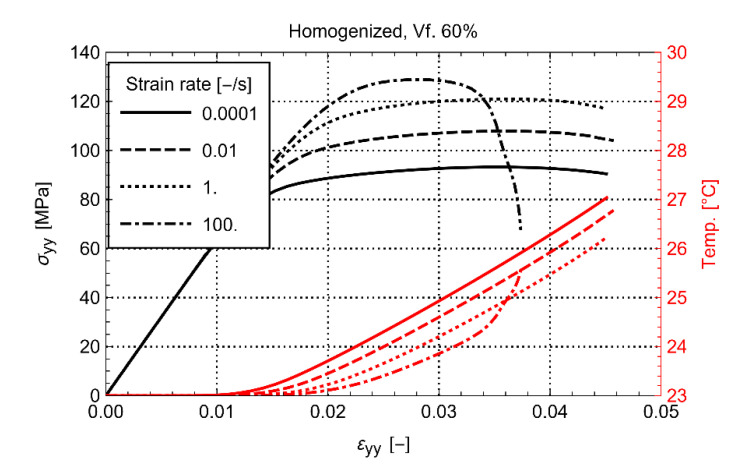
Effect of strain rate on homogenized stress-temperature-strain for Vf. 60%.

It is remarkable that considerable softening is also observed for the lowest strain rate of $10^{-4}\;s^{-1}$, despite that the global heating of the material is quite limited. The latter suggests that the effect of the local heating of the matrix between the fibers can be significant, even at quasi-static strain rate. The phenomenon becomes more prominent with increasing volume fraction and increasing strain rate. For example, in Figure 7a, the loss is moderate for every strain rate at a fiber volume fraction of 30%. At the same time, as shown in Figure 7d, the response is catastrophic for the load carrying capability of the material at the volume fraction of 60% and the highest strain rate. Despite the loss of strength in this case, it is observed that the homogenized temperature at the strain rate of $100\;s^{-1}$ is just a few degrees (Figure 8). To result in such a significant stiffness loss, the local heating of the matrix must, therefore, be significant.

**Figure 9:**
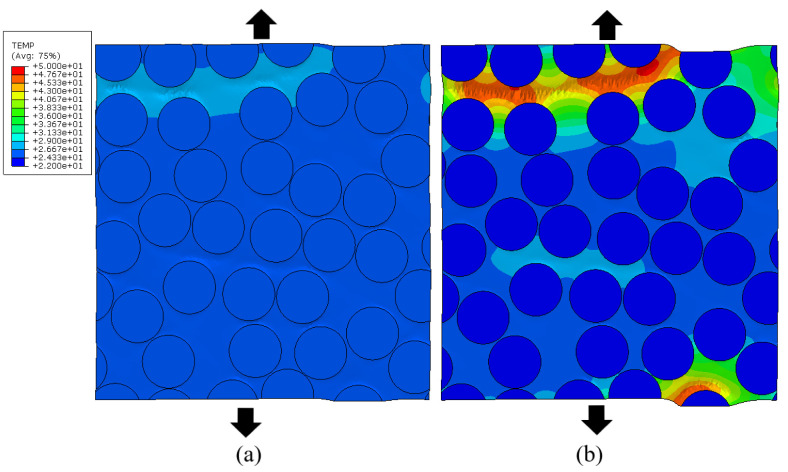
Temperature distribution for the RVEs with Vf. 60% at a strain of 0.037 [-]. strain rate 1. [s^−1^] (**a**) and 100. [s^−1^] (**b**).

The latter is confirmed in Figure 9, where the temperature distribution in the matrix for the RVE with the volume fraction of 60% for the same homogenized strain level is compared between the strain rate of $1\;s^{-1}$ and $100\;s^{-1}$. It can clearly be seen that, for the high strain rate case, a horizontal band with elevated temperature in the matrix is generated. In this band, the matrix has significantly softened, leading to unloading of the other parts in the RVE, while the hot band takes up the remaining deformation.

This result shows that not only microcracks but also matrix softening from self-heating might produce a substantial loss of material strength. This might happen, especially in the case when a matrix material with significant self-heating behavior is used. For the PA6 matrix material used in this work, self-heating is confirmed in [45]. For engineering purposes, it can be interesting to determine the threshold where local thermal softening induces premature failure in the UD FRP. For this, one would have to match the strain rate at which thermal softening induces premature failure in the pure matrix to the strain rate occurring locally in the matrix pockets of the UD FRP. Although the authors stress that, in reality, one would also have to account for the occurrence of matrix cracks, this method can give a fast indication as to whether thermal softening should be taken into account for the particular application.

### 4.4. Effect of Interface Conductivity

The effect of the conductivity of the fiber-matrix interface is investigated by modifying its value between $0\;Wm^{-1}K^{-1}$ and $1.256\;Wm^{-1}K^{-1}$. The latter corresponds to the transverse thermal conductivity of the carbon fibers (Table 1). This range also covers the thermal conductivity of the matrix ($0.125\;Wm^{-1}K^{-1}$).

**Figure 10:**
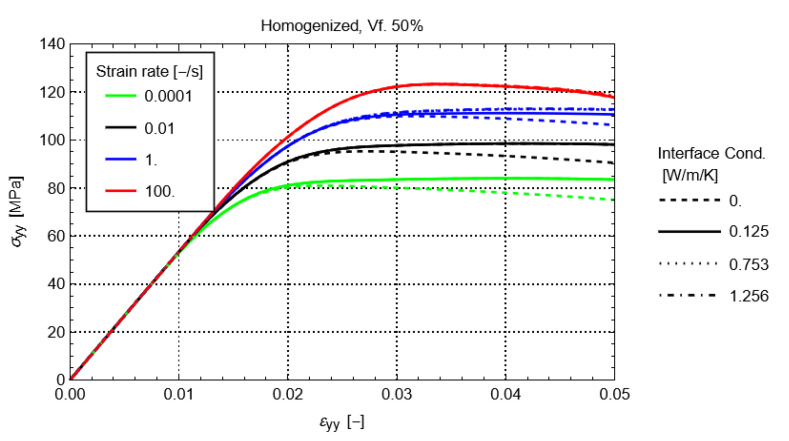
Effect of fiber-matrix interface conductivity on homogenized stress-strain at multiple strain rates for a volume fraction of 50%.

Figure 10 shows the homogenized stress-strain response of the RVE for the volume fraction of 50%. The results for the other volume fractions are similar. In the figure, it can be seen that the thermal conductivity of the interface has a limited effect on the homogenized response. Only when the thermal conductivity of the interface is lower than the one from the matrix, a difference can be observed for strain rates lower than $1\;s^{-1}$. An additional softening is observed, which can be explained by the obstruction of heat flow to the fiber material, resulting in an elevated heating and softening of the matrix. The effect increases with an increasing volume fraction of the material. In reality, it is unlikely that the thermal conductivity of the fiber-matrix interface would be lower than that of the matrix. This would only occur if there were defects present, e.g., air pockets or debonding, which can obstruct heat flow. The latter is not expected in a well-designed and undamaged FRP material. Therefore, the effect of the conductivity of the fiber-matrix interface is not of primary concern in the thermomechanical modeling of RVEs.

## 5. Conclusions

In this work, the effect of self-heating from the visco-plastic behavior of the matrix material in continuous fiber-reinforced thermoplastic composites is investigated using micromechanical modeling. A representative RVE of Carbon/PA6 UD composite material is constructed with the Finite Element Method. Several random fiber distributions and volume fractions are generated. Hereby, the PA6 matrix constitutive behavior is modeled with an improved representation of the double-yield phenomenon considering a temperature-dependent visco-plastic response, including self-heating and thermal softening effects. The micro RVE model accounts for both mechanical and thermal periodic boundary conditions. The effect of self-heating is investigated by applying several strain rates and modifying the parameters that govern the self-heating behavior and the interface conductivity. In summary, the following conclusions can be drawn:

- 32 fibers are sufficient to provide a converged homogenized thermomechanical response of an RVE with volume fractions ranging between 30% and 60%.
- The double-yield phenomenon, observed in stress-strain curves of semi-crystalline polymers, is concealed in the homogenized behavior of the composite material.
- Localized highly strained matrix regions can be present with significant self-heating. Although they do not notably increase the homogenized temperature of the RVE, the self-heating can be significant, leading to premature plastic-like failure of the material. The lack of visibility in the homogenized temperature also indicates that this phenomenon will be very hard to measure experimentally since it happens on a microscopic scale.
- The effect of the conductivity of the fiber-matrix interface is limited to cases where the conductivity is lower than the conductivity of the matrix. Since this is an unlikely scenario, the influence of this parameter is of secondary importance.

This work shows that matrix self-heating and its associated softening can have a significant influence on the mechanical behavior of fiber reinforced thermoplastic materials. Up to now, this effect has not been thoroughly investigated in relation to strain rate, volume fraction and thermal conductivity of the fiber-matrix interface. This work provides a further basis for the investigation of the macroscopic behavior with micromechanical modeling for other loading directions, such as shear loading, and including additional phenomena, such as fiber-matrix debonding, fiber failure and matrix failure.

The results of this study can be limitedly projected on UD FRP with other combinations of fibers (glass, boron, polyimide) and matrix (PP, PET). It is expected that, for fibers with a lower transverse thermal conductivity than carbon fibers, the self-heating effect is more significant because the generated heat in the matrix will be more confined. The fiber diameter might also have an effect. For this, the authors cannot foresee the outcome. The latter will be the subject of future work.

## Figures and Tables

**Table 1:** Carbon fiber mechanical and thermal properties.

Mechanical [26]		Thermal [36]	
$E_{11}$ [GPa]	276.0	$k_{11}$ [Wm^−1^K^−1^]	10.2
$E_{22}=E_{33}$ [GPa]	15.0	$k_{22}=k_{33}$ [Wm^−1^K^−1^]	1.256
$G_{12}=G_{13}\;$[GPa]	15.0	ρ [kg m^−3^]	1800
$G_{23}$ [GPa]	5.8	$c_{p}$ [J kg^−1^ K^−1^]	750
$\nu_{12}=\nu_{13}$ [-]	0.01		
$\nu_{23}$ [-]	0.3		

**Table 2:** Properties of PA6 matrix.

Mechanical		Thermal [31]	
$E_{ref}$ [GPa]	2.6	k [Wm^−1^K^−1^]	0.25
$\theta_{ref}$ [K]	296.15	$c_{p}$ [Jkg^−1^K^−1^]	1700
β [K^−1^]	0.0072	ρ [kg m^−3^]	1200
ν [-]	0.39		
${\dot{\varepsilon }}_{o}$ [1/s]	3.55 × 10^11^		
A [K MPa^−1^]	104.0		
m [-]	0.80		
$s_{0}$ [MPa]	184.2		
$s_{1}$ [MPa]	195.6		
$s_{2}$ [MPa]	192.6		
$s_{3}$ [MPa]	232.6		
$h_{1}$ [MPa]	32,350.9		
$h_{2}$ [MPa]	14,827.2		
$h_{3}$ [MPa]	900.0		
${\bar{\varepsilon }}_{p}$ [-]	0.00905		
${\bar{\varepsilon }}_{c}$ [-]	0.03205		
f [-]	0.30		

## Data Availability

Provided upon request.
